# Optogenetic Recruitment of Dorsal Raphe Serotonergic Neurons Acutely Decreases Mechanosensory Responsivity in Behaving Mice

**DOI:** 10.1371/journal.pone.0105941

**Published:** 2014-08-22

**Authors:** Guillaume P. Dugué, Magor L. Lörincz, Eran Lottem, Enrica Audero, Sara Matias, Patricia A. Correia, Clément Léna, Zachary F. Mainen

**Affiliations:** 1 Champalimaud Neuroscience Programme, Champalimaud Center for the Unknown, Lisbon, Portugal; 2 Institut de Biologie de l'Ecole Normale Supérieure, Centre National de la Recherche Scientifique (CNRS) UMR8197, Institut National de la Santé et de la Recherche Médicale (INSERM) U1024, Paris, France; 3 MTA-SZTE Research Group for Cortical Microcircuits, Department of Physiology, University of Szeged, Szeged, Hungary; McGill University, Canada

## Abstract

The inhibition of sensory responsivity is considered a core serotonin function, yet this hypothesis lacks direct support due to methodological obstacles. We adapted an optogenetic approach to induce acute, robust and specific firing of dorsal raphe serotonergic neurons. *In vitro*, the responsiveness of individual dorsal raphe serotonergic neurons to trains of light pulses varied with frequency and intensity as well as between cells, and the photostimulation protocol was therefore adjusted to maximize their overall output rate. *In vivo*, the photoactivation of dorsal raphe serotonergic neurons gave rise to a prominent light-evoked field response that displayed some sensitivity to a 5-HT1A agonist, consistent with autoreceptor inhibition of raphe neurons. In behaving mice, the photostimulation of dorsal raphe serotonergic neurons produced a rapid and reversible decrease in the animals' responses to plantar stimulation, providing a new level of evidence that serotonin gates sensory-driven responses.

## Introduction

The influences of central serotonin (5-hydroxytryptamine or 5-HT) impact a wide range of brain functions, from the control of autonomic responses [1], [2] to the regulation of complex emotional behaviors [3], [4]. These diverse influences may be systematized by considering possible core neurophysiological functions. Among these, serotonergic neuromodulation has long been implicated in the inhibition of sensory responsivity [5], [6], an idea chiefly supported by gain-of-function experiments. Pharmacological enhancement of 5-HT function inhibits primary afferent neurotransmission *in vitro* [7], [8], dampens sensory and nociceptively-evoked firing *in vivo* [9]-[11], [12] and decreases acoustic startle responses and their pre-pulse inhibition [6], [13]. Similarly, electrical microstimulation of the dorsal raphe nucleus (DRN), one of the largest sources of ascending 5-HT projections [14], reduces forebrain sensory and nociceptively-evoked activity [9], [11], [12], [15]–[17] and elevates vocalization thresholds to noxious stimuli [18].

Despite these observations, technical limitations have impeded a deeper understanding of the underlying mechanisms. Pharmacological upregulation of 5-HT pathways neither mimics phasic 5-HT release nor takes into account the effect of co-released substances [19], [20], and may exhibit paradoxical effects due to autoreceptor-mediated negative feedback [21] and drug-induced plastic changes [22]. Electrical stimulation, while spatially and temporally precise, can produce non-specific effects by activating non-5-HT neurons and fibers-of-passage [18]. To overcome these technical limitations, we optimized and validated a direct and specific optogenetic stimulation of DRN serotonergic neurons in mice. We then employed this strategy to test whether transient and specific activation of DRN 5-HT neurons in behaving mice can indeed interfere with sensory responsivity in a simple test of mechanosensitivity.

## Materials and Methods

All procedures were performed in accordance with the Champalimaud Foundation Ethics Committee guidelines, and approved by the Portuguese Veterinary General Board (Direcção Geral de Veterinária, approval ID 014315). Detailed methods are available in Supporting Information S1.

### Viral transduction of DRN neurons

Adeno-associated viral vectors carrying a Cre-activated ChR2-YFP expression cassette (AV-1-20298P, University of Pennsylvania, 10^13^ GC/mL) were stereotaxically injected (volume: 1.0–1.2 µL) into the DRN of adult (8–16 weeks) transgenic SERT-Cre or wild-type mice. The SERT-Cre mouse line [23] was obtained from the Mutant Mouse Regional Resource Centers (stock number: 017260-UCD). For behavioral testing, an optical fiber (200 µm, 0.37 NA) housed inside a connectorized implant (M3, Doric Lenses) was positioned over the DRN after the injection. The detailed coordinates of the injection and fiber placement are provided in Supporting Information S1.

### Immunohistochemistry

Animals were anesthetized with 5% chloral hydrate (500 mg/kg) and perfused with 4% PFA. After cryoprotection (in 30% sucrose), coronal sections (12 µm) were cut in a cryostat, mounted on glass slides and incubated in 10 mM citrate buffer (pH 6.0, 0.05% Tween) at 96°C for 10 min. After washing with TBS, sections were preincubated for 1 hour at room temperature in a blocking solution containing 5% fetal bovine serum, 2% bovine serum albumin and 0.25% Triton X-100 diluted in TBS. Section were incubated overnight at 4°C in a blocking solution containing a mouse monoclonal anti-TPH antibody (T0678, Sigma-Aldrich, 1∶400) and a rabbit polyclonal anti-GFP antibody (A6455, Life Technologies, 1∶400). Sections were then incubated for 1 h at room temperature in a blocking solution containing an Alexa Fluor 488 goat anti-rabbit antibody and an Alexa Fluor 594 goat anti-mouse antibody (Life Technologies, diluted 1∶1000). ChR2-YFP- and TPH-positive cell bodies were counted on projections of 20 confocal images taken every 0.5 µm.

### *In vitro* recordings and photostimulation of DRN neurons

DRN slices were prepared from SERT-Cre mice aged 8–20 weeks (2–4 weeks post-infection). DRN cells were recorded in the loose cell-attached (n = 4 non-fluorescent and 28 fluorescent cells) and whole-cell configurations (n = 3 non-fluorescent and 6 fluorescent cells). Cells were illuminated using a 465 nm LED coupled to a 200 µm 0.37 NA optical fiber (Doric Lenses) held at 34° at a fixed distance above the slice. For each cell, the incident irradiance was calculated by dividing the power measured at the fiber tip by the area of the illuminated zone.

### Blue light propagation in the DRN

The spread of blue light in the DRN was assessed in freshly dissected brains hemisected in the sagittal plane. The blocks of tissue were immersed in PBS and the plane of cut was imaged from the top. Blue light was delivered through an LED-coupled optical fiber positioned next to the dorsal aspect of the DRN. The detailed procedure is available in Supporting Information S1.

### Fluorescence mapping of ChR2-EYFP-expressing DRN neurons and recordings of local photoevoked activity *in vivo*

Custom optrodes were assembled by gluing a 200 µm 0.37 NA multimode optical fiber (BFL37-200, Thorlabs) onto a platinum microelectrode (0.8–1.0 MΩ at 1 kHz, FHC). Light pulses were generated using a laser beam (473 nm, 100 mW DPSS laser, Laserglow) gated by a mechanical shutter (VS14S2ZM1, Uniblitz) and attenuated by a set of neutral density filters. The tissue fluorescence collected by the optical fiber was monitored through a dichroic mirror using a CCD camera.

### Von Frey test

17 SERT-Cre mice and 17 wild type littermates (control group) were infected with the Cre-dependent viral vector and implanted with connectorized optical fibers. After recovery (one week), animals were allowed to habituate to the testing box (a 9×7×14 cm Plexiglas box placed on an elevated metal mesh platform) 5 minutes per day for 5 days, during which the 4.0 g filament was applied 5 times per hind paw. In the testing phase, filaments ranging 0.4–8 g were applied consecutively in an ascending fashion (each filament was applied 5 times successively to the right and left hind paws). This procedure was repeated 3 times per session: before (“baseline”), in conjunction with (“stim”) and after (“recovery”) photostimulation. The “stim” and “recovery” blocks were separated by a 5 minutes delay. In the “stim” condition, filament application was restricted to the last three quarters of a 12 s photostimulation train (10 ms, 20 Hz, 318 mW.mm^−2^ at the fiber tip). The 3 s delay between photostimulation onset and first filament application are meant to allow the establishment of a potentially slow serotonergic neuromodulatory mechanism.

## Results

### *In vitro* photostimulation of virally transduced dorsal raphe 5-HT neurons

**Figure 1:**
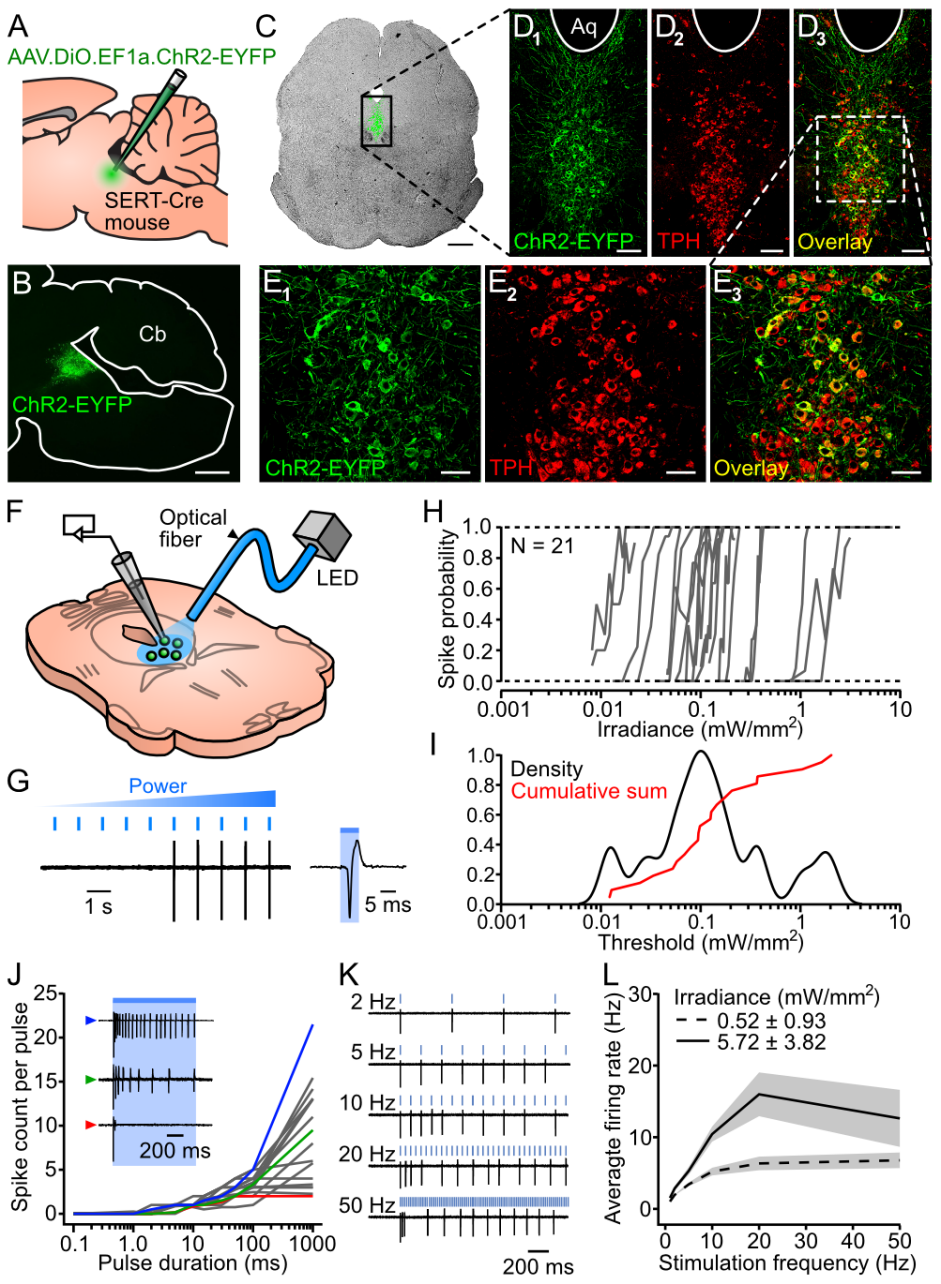
Specificity of ChR2-YFP expression in DRN 5-HT neurons and efficiency of photostimulation *in vitro*. ***A***, Schematics of viral injections. ***B***, Fluorescence picture of a parasagittal slice showing restricted ChR2-YFP expression in the DRN. ***C***, Superimposed fluorescence and brightfield images of a ChR2-YFP-expressing coronal slice. Bar: 500 µm. ***D_1–3_***, Confocal pictures of the area delimited by a black rectangle in *C*. Aq: aquaduct. Bar: 100 µm. ***E_1–3_***, Magnified view of the area delimited by a white rectangle in *D* ***_3_***. Bar: 50 µm. ***F***, Schematics of patch-clamp recordings in DRN slices. ***G***, Left: example of a loose cell-attached recording illustrating the protocol used to assess photostimulation thresholds (PTs). Right: blow-up of the trace showing a single photoevoked spike. ***H***, Spike probability versus incident irradiance for 21 cells. ***I***, Kernel density estimate of the distribution of logarithmically-scaled PTs (black) and superimposed normalized cumulative sum (red). ***J***, Spike count per pulse versus pulse duration for 17 cells at twice their PT. Inset: representative firing profiles of 3 different cells identified in the graph by colored arrowheads. ***K***, Response of a ChR2-YFP cell to trains of repeated light pulses (6 ms) at various frequencies (irradiance set at twice its PT). ***L***, Average firing rate (± SEM, shaded area) during a 5 s train versus photostimulation frequency at twice the PT (dashed line; n = 29, 28, 25 and 18 cells for 1–2, 5–10, 20 and 50 Hz respectively) and at a higher irradiance (∼5 mW·mm^−2^; solid line, n = 14 and 13 cells for 1–20 and 50 Hz respectively).

To overcome the limitations of previous studies and explore the specific functions of DRN 5-HT projections, we targeted channelrhodopsin-2 fused to YFP (ChR2-YFP) to DRN 5-HT neurons using a viral expression strategy. Adeno-associated viruses (AAVs) carrying a Cre-activated expression cassette for ChR2-YFP were injected into the DRN of SERT-Cre mice [23] (Fig. 1*A* and *B*). The specificity of this approach was assessed using anti-tryptophan hydroxylase (TPH) immunocytochemistry (Fig. 1*C*–*E*). TPH immunoreactivity was observed in 93.9±2.0% of ChR2-YFP cells (n = 3 brains, respectively 287/308, 293/305 and 507/549 counted ChR2-YFP-positive cells also positive for TPH), confirming that this method yields 5-HT neuron-specific expression. The photosensitivity of ChR2-YFP neurons was assessed in acute DRN slices (Fig. 1*F*–*G*). Non-fluorescent cells displayed no photoevoked spike or current in response to the highest irradiance tested (1.5–10.4 mW·mm^−2^, n = 7). In contrast, all ChR2-YFP cells fired in response to brief (6 ms) light pulses (n = 34), with an average photostimulation threshold (PT) of 0.26±0.47 mW·mm^−2^ (calculated for a subset of n = 21 cells; Fig. 1*G*–*I*). Longer pulses (1 s) at twice the PT evoked firing profiles ranging from non- to fully-inactivating (Fig. 1*J*). Firing evoked by repeated photostimulation (6 ms pulses at 1–50 Hz) generally adapted over time (Fig. 1*K*). During 5 s photostimulation trains, ChR2-YFP cells could reliably (spike probability>0.85) follow up to 2 Hz at twice their PT and up to 10 Hz at a higher irradiance (∼5 mW·mm^−2^). However the maximal average firing rates for these two irradiances (6.8±4.6 Hz, n = 18 and 16.0±11.4 Hz, n = 14) were attained with 50 and 20 Hz photostimulations respectively (Fig. 1*L*). Therefore irradiances one order of magnitude above PT are required to induce firing rates comparable to those observed in behaving animals during phasic episodes of increased DRN firing [24]–[26].

### *In vivo* photostimulation of dorsal raphe 5-HT neurons

**Figure 2:**
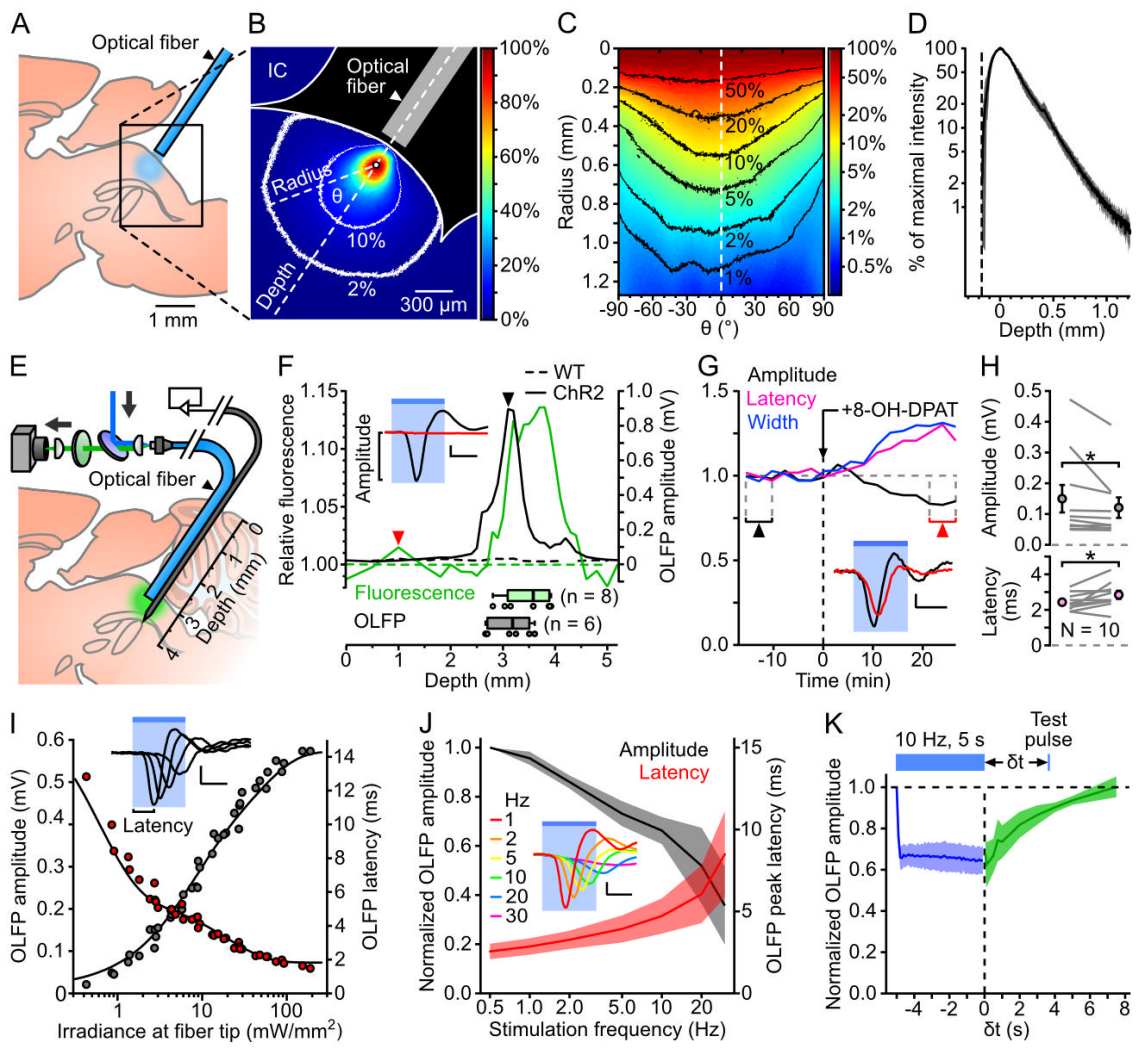
Photostimulation of DRN 5-HT neurons *in vivo*. ***A***, Schematics of the experiment used to assess the spread of blue light in the DRN. ***B***, Example image of DRN illumination. The intensity is color-coded relative to the pixel of maximal intensity (white dot). The 10 and 2% contour lines are shown in white. IC: inferior colliculus. ***C***, Circular profile plot calculated from *B*, showing pixel intensities along concentric lines centered on the brightest pixel. ***D***, Average intensity profile (± SD, shaded area; n = 3 brains) along the fiber axis. The depth and intensities are calculated relative to the brightest pixel. ***E***, Schematics of the setup used to map YFP fluorescence and photoevoked firing *in vivo*. ***F***, Example of a combined electrophysiological and fluorescence mapping experiment in a ChR2-YFP-expressing SERT-Cre (ChR2, solid lines) and a wild-type (WT, dotted lines) mouse. Top: normalized fluorescence (green, left axis) and OLFP amplitude (black, right axis) profiles plotted against the optrode position. Bottom: boxplots showing the optrode position at the point of maximal fluorescence (green, n = 8 mice) and largest OLFP amplitude (grey, n = 6 mice). Inset: average OLFP for the ChR2 mouse at the locations marked by the arrowheads. ***G***, Example showing the time course of the effect of the 5-HT1A receptor agonist 8-OH-DPAT on the OLFP amplitude (black), latency (purple) and width (blue). Bin: 158 s. Inset: average OLFP before (black) and after (red) 8-OH-DPAT injection (taken from the periods indicated by arrowheads). ***H***, Top: OLFP amplitude before and after 8-OH-DPAT injection (mean ± SD: 150±121 µV and 121±105 µV respectively, n = 10). Bottom: OLFP peak latency before and after 8-OH-DPAT injection (mean ± SD: 2.4±0.5 ms and 2.8±0.7 ms, n = 10). Changes are significant in both cases (*P* = 0.037 and *P* = 0.030 respectively, paired Wilcoxon rank sum test). Error bars represent the SEM. ***I***, Example illustrating the dependence of the OLFP amplitude (grey points) and latency (red points) on the irradiance at the fiber tip. The sum of two exponential functions was fitted to each curve (black lines). Inset: superimposed OLFPs for 0.2, 0.7, 1.6 and 6.0 mW·mm^−2^. ***J***, Normalized average (± SD, shaded area) OLFP amplitude (black, left axis) and latency (red, right axis) versus photostimulation frequency (n = 7 mice). ***K***, Average OLFP amplitude recovery curve (green; ± SD, shaded area) assessed by delivering test pulses at variable intervals (δt) after 10 Hz, 5 s trains of 6 ms light pulses (n = 4 mice). Blue: average amplitude during the trains.

In order to estimate how far from the fiber tip ChR2-YFP neurons could be recruited *in vivo*, we measured the spread of blue light in the DRN of hemisected brains (Fig. 2*A and B*; see Methods). Light intensity decayed in an exponential fashion away from the fiber tip, with a space constant of 199±35 µm (n = 3; Fig. 2*C* and *D*). The volume of tissue receiving more than 2% of the irradiance at the brightest point extended 1.65±0.21 mm, >1.00 and 1.23±0.10 mm in the antero-posterior, medio-lateral and fiber axes respectively (n = 3). We then probed the response of DRN 5-HT neurons *in vivo* by advancing a custom optrode toward the DRN of anaesthetized animals, while intermittently shining light pulses. The tissue fluorescence (Fig. 2*E*) displayed a peak 3.48±0.43 mm below the cerebellar surface (n = 8), which correlated spatially with a prominent multiphasic photoevoked potential (OLFP, optically-evoked local field potential; Fig. 2*F*). Systemic administration of the 5-HT1A receptor agonist 8-OH-DPAT, a drug commonly employed to inhibit 5-HT cell firing by activating local autoreceptors [21], blunted the OLFP amplitude and prolonged its latency (Fig. 2*G and H*). The OLFP peak amplitude and latency displayed a graded dependence on the irradiance at the fiber tip (Fig. 2*I*), approaching saturation (slope<10 µV/mW·mm^−2^) at 117±34 mW.mm^−2^ (n = 9). This dependence could be described as the sum of two exponential functions (*τ*_fast_ and *τ*_slow_ of 7.4±6.0 and 53.5±18.7 mW.mm^−2^ accounting for 46% and 54% of the amplitude respectively; *τ*_fast_ and *τ*_slow_ of 3.1±5.2 and 29.6±26.2 mW.mm^−2^, accounting for 70% and 30% of the latency respectively; n = 9). The OLFP waveform also rapidly adapted to trains of repeated photostimulation (reduced amplitude and extended latency with increased stimulation frequency, Fig. 2*J*), an effect potentially mediated by a combination of factors such as partial inactivation of voltage-dependent sodium channels, ChR2 desensitization, a build-up of afterhyperpolarization currents or the activation of 5-HT1A autoreceptors. The OLFP recovery kinetics, assessed for 10 Hz trains, had a time constant of 2.9±0.5 s (n = 4; Fig. 2*K*), and full recovery was observed after 8 seconds.

### Decreased mechanosensory responsivity of behaving mice during acute dorsal raphe 5-HT neurons photostimulation

**Figure 3:**
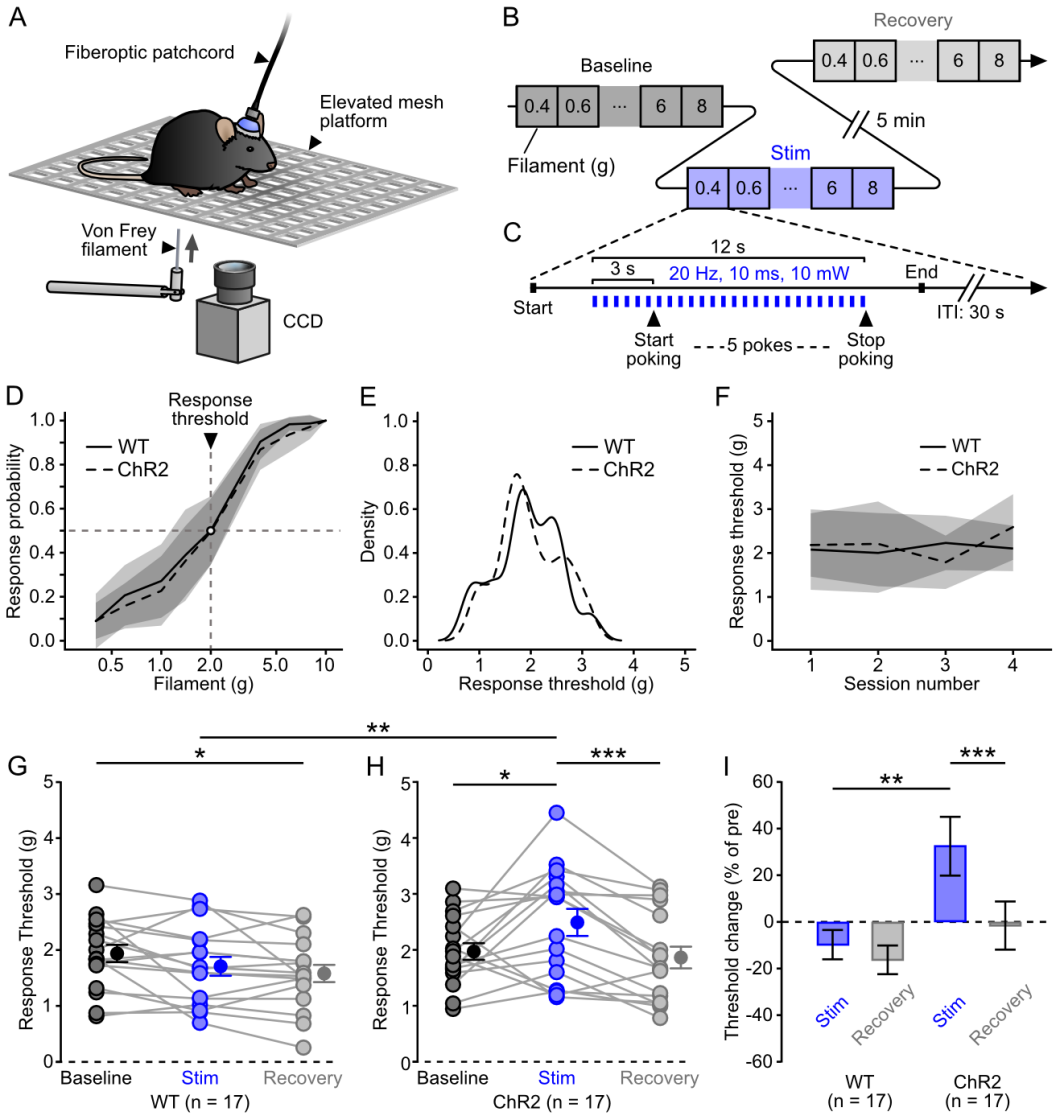
Decreased responsivity to mechanical stimuli during DRN 5-HT neurons photostimulation. ***A***, Schematics of the experiment. ***B***, Structure of a session. ***C***, Structure of a trial in the “stim” block. The filament was applied to the hindpaw during a 12 s train of light pulses. ***D***, Average baseline response probability curves for control (infected WTs, solid line, n = 17) and ChR2 (dashed line, n = 17) mice. ***E***, Kernel density estimates of the baseline threshold distribution for control (WT, solid line) and ChR2 (dashed line) mice. ***F***, Average baseline threshold across sessions for control (WT, solid line) and ChR2 (dashed line) mice. ***G–H***, Thresholds for all control and ChR2 mice in the baseline (dark grey), “stim” (blue) and recovery (light grey) blocks. *: *P*<0.05 (paired Wilcoxon rank sum test); ***: *P*<0.001 and **: *P*<0.01 (unpaired Wilcoxon rank sum test). (*I*) Threshold change for control and ChR2 mice in the “stim” (blue) and recovery (light grey) blocks. ** *P*<0.01 and *** *P*<0.001 (paired and unpaired Wilcoxon rank sum test respectively). Shaded areas and error bars represent the SD in all panels.

Having characterized the specificity and efficacy of the photostimulation of DRN 5-HT neurons, we then tested this methodology in behaving animals using a classical test of mechanical sensitivity, the von Frey assay [27] (Fig. 3*A*). In this test, a series of calibrated Nylon filaments (von Frey hairs) of ascending stiffness are applied to the plantar surface of the hind paws while monitoring the animal's withdrawal response. Groups of SERT-Cre (ChR2) and littermate wildtype (WT) control mice (n = 17 for both) were infected with the same viral vector. Both groups had the same sex ratio (9 males, 8 females) and were implanted with optical fibers positioned over the DRN. After a habituation period of 5 days (see Methods), animals were tested during 3 to 4 sessions, with a maximum of 1 session per day. Each session was divided in 3 blocks, designed to test the animal's sensitivity prior to (“baseline”), in conjunction with (“stim”) and after (“recovery”) photostimulation (Fig. 3*B*). For each block, a psychometric curve (response probability versus filament) was calculated and the response threshold was taken as the interpolated filament value corresponding to a response probability of 0.5. Baseline responsivity to ascending forces applied with von Frey hairs (0.4–8 g) to the hind paws did not differ between SERT-Cre and WT mice (Fig. 3*D*–*E*), with average response thresholds of 1.93±0.63 g and 1.97±0.61 g, respectively (*P* = 0.97, unpaired Wilcoxon sum rank test). However in the presence of concomitant photostimulation (a 12 s train of 10 ms light pulses at 20 Hz, ∼300 mW·mm^−2^ at the fiber tip, Fig. 3*B and C*), response thresholds were significantly higher for ChR2 animals than WT controls (2.49±1.00 g vs 1.71±0.69 g, *P* = 0.0076, unpaired Wilcoxon sum rank test). This difference was no longer observed in the “recovery” block, 5 minutes after the last photostimulation train (Fig. 3*G* and *H*). The effect observed in ChR2 mice during photostimulation corresponded to a significant threshold elevation of 32.5±52.0% (Fig. 3*I*) which counteracted the slight sensitization observed in WT animals (Fig. 3*G*). This result shows that acute DRN 5-HT photostimulation induces a transient and fully reversible decrease in responsivity to plantar stimulations in behaving mice.

## Discussion

The initial optogenetic approaches used to study DRN functions employed non-specific promoters [28], [29] or targeted local or distal neurons presynaptic to 5-HT neurons [28], [30]. Recently, specific optogenetic stimulation of DRN 5-HT neurons has been achieved using transgenic mouse lines [31], [32] or viral injections [33] but these studies have not provided a detailed account of optimal photostimulation parameters. Here we devoted substantial efforts to optimizing the yield of direct and specific photostimulation of DRN 5-HT neurons. ChR2-EYFP-expressing cells were sensitive to low irradiance *in vitro* (<1 mW·mm^−2^, Fig. 1*H*–*I*). However high irradiances (>100 mW·mm^−2^ at fiber tip) were necessary to saturate the photoevoked local field potential *in vivo*, a measure that might prove useful for optimally positioning optical fibers and assessing levels of ChR2-YFP expression in target structures. Using irradiance of >250 mW·mm^−2^ at the fiber tip, we estimated that the entire DRN received irradiances over 5–6 mW·mm^−2^, a value at which the output of ChR2-EYFP neurons could be maximized (up to 16 Hz) *in vitro* by using 20 Hz stimulations, despite their strong frequency-dependent adaptation. These parameters are appropriate to attempt to mimic episodes of increased DRN activity, which occur in association with a variety of behavioral conditions such as oro-buccal movements [24] and defensive encounters [26], and in relation to reward outcome [34] and waiting for delayed rewards [25]. Such episodes typically last several seconds, during which the activity of DRN neurons can peak up to 10–20 Hz.

Our protocol for DRN 5-HT neuron photostimulation in behaving mice (20 Hz for 12 seconds) evoked robust decreases in behavioral responses to hind paw stimulation. Previous observations have shown that chronic elevation of 5-HT levels can increase response thresholds in rodent models of mechanical allodynia [35]–[37]. Our result extends this observation by showing that a similar effect can be reversibly induced on a faster timescale in non-pathological conditions by recruiting DRN 5-HT neurons. Whether the threshold calculated using von Frey filaments in naïve animals is a measure of sensory or nociceptive sensitivity is still a debated question [27]. Therefore the question whether the stimulation of DRN 5-HT neurons in our conditions primarily acts upon sensory or nociceptive pathways remains open. Nevertheless, our result helps to resolve the ambiguity of previous gain-of-function experiments testing the influence of DRN output by directly showing that DRN 5-HT neurons can indeed tone down the influence of sensory and/or nociceptive inputs, as opposed to what has been recently observed in zebrafish [38].

Given the projection pattern of DRN 5-HT cells [14], this effect is likely to be mediated by the modulation of anterior structures, as suggested by evidence highlighting a role for 5-HT in the modulation of thalamic [11], [12], [16], [39] and cortical [5], [10] sensory and nociceptive responses. It is not unlikely that other co-released substances may play a role in the observed effect. In particular, a recent study has shown that the glutamatergic phenotype of certain 5-HT neurons seems to be partly responsible for the effects produced by the photostimulation of DRN 5-HT neurons on reward-related behaviors [33].

More refined targeting strategies, e.g. retrograde infection [40] or intersectional genetics [41], will allow the assessment of contributions of specific sub-populations of DRN 5-HT neurons [14]. Overall these results provide a new level of evidence for the involvement of DRN 5-HT neurons in gating the access of sensory inputs to behavioral output, a key physiological role which will help constrain larger-scale theories of 5-HT function.

## Supporting Information

Figure S1 **Photostimulation of DRN 5-HT neurons** ***in vitro*** **.**
(TIF)

Figure S2 **Light propagation in the DRN.**
(TIF)

Supporting Information S1 Table S1: Von Frey filaments force scale.
(DOCX)
