# Supplementary figures and images for: Optogenetic Recruitment of Dorsal Raphe Serotonergic Neurons Acutely Decreases Mechanosensory Responsivity in Behaving Mice
Source: PLoS One. 2014 Aug 22;9(8):e105941. doi: 10.1371/journal.pone.0105941 (PMC4141837; doi:10.1371/journal.pone.0105941)

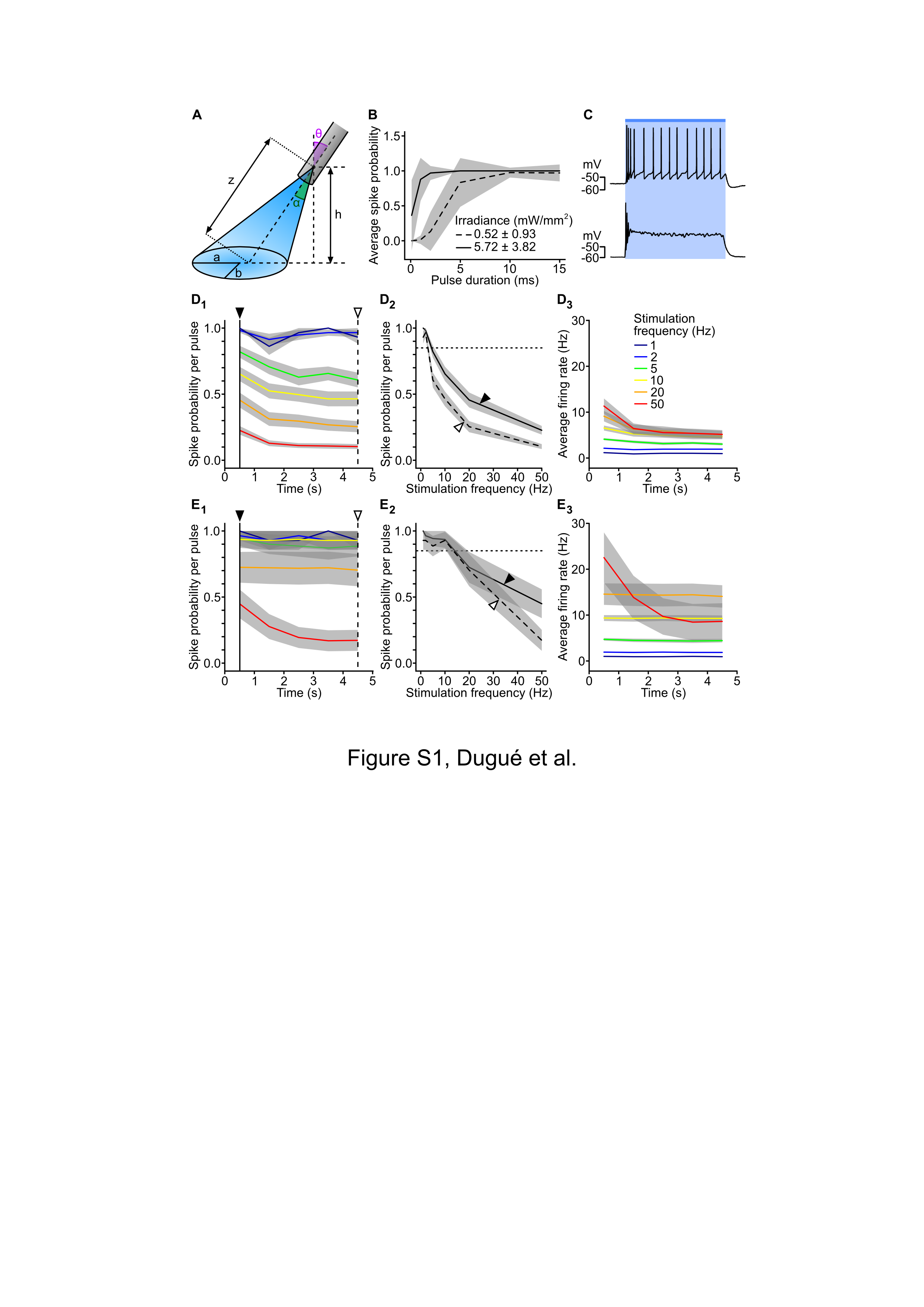

Supplement: Figure S1 — Photostimulation of DRN 5-HT neurons in vitro . (TIF) [file pone.0105941.s001.tif]

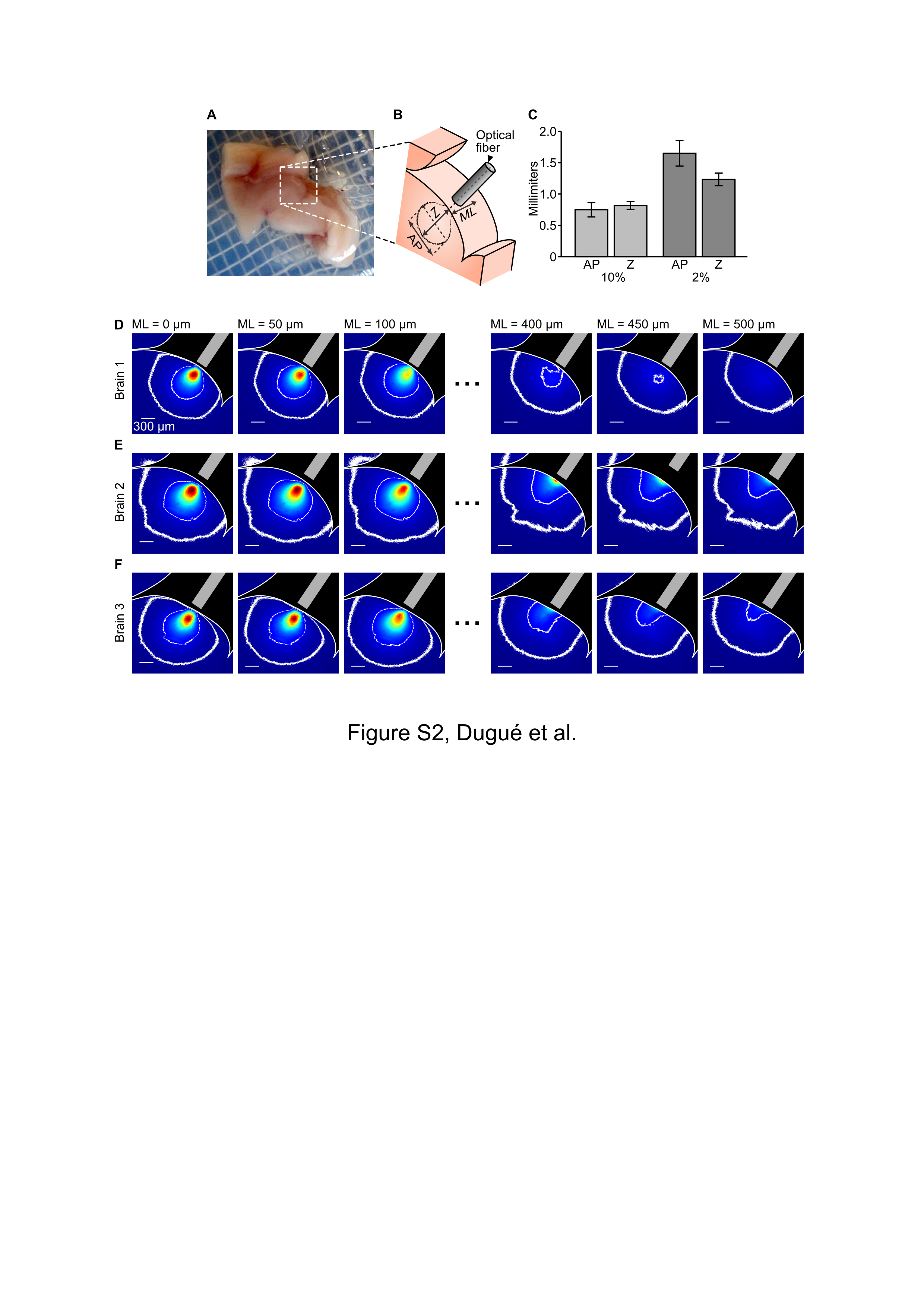

Supplement: Figure S2 — Light propagation in the DRN. (TIF) [file pone.0105941.s002.tif]
