# Supplementary material for: Optogenetic Recruitment of Dorsal Raphe Serotonergic Neurons Acutely Decreases Mechanosensory Responsivity in Behaving Mice
Source: PLoS One. 2014 Aug 22;9(8):e105941. doi: 10.1371/journal.pone.0105941 (PMC4141837; doi:10.1371/journal.pone.0105941)
Supplement: Supporting Information S1 — Table S1: Von Frey filaments force scale. (DOCX) [file pone.0105941.s003.docx]

**SUPPORTING INFORMATION**

**Optogenetic recruitment of dorsal raphe serotonergic neurons acutely decreases mechanosensory responsivity in behaving mice**

Guillaume P. Dugué^a,b,2^, Magor L. Lörincz^a,c^, Eran Lottem^a^, Enrica Audero^a^, Sara Matias^a^, Patricia A. Correia^a^, Clément Léna^b,1^ & Zachary F. Mainen^a,1,2^

^a^Champalimaud Neuroscience Programme, Champalimaud Center for the Unknown, Lisbon, Portugal.

^b^Institut de Biologie de l'Ecole Normale Supérieure, Centre National de la Recherche Scientifique (CNRS) UMR8197, Institut National de la Santé et de la Recherche Médicale (INSERM) U1024, Paris, France.

^c^MTA-SZTE Research Group for Cortical Microcircuits, Department of Physiology, University of Szeged, Szeged, Hungary.

^1^Both authors contributed equally to this work.

^2^Corresponding authors:

Guillaume P. Dugué Zachary F. Mainen

Institut de Biologie de l’Ecole Normale Supérieure Champalimaud Neuroscience Programme

46, rue d’Ulm Champalimaud Center for the Unknown

75005 Paris, France Avenida Brasilia s/n

Tel : +33 144323730 1400-038 Lisboa, Portugal

Email: gdugue@biologie.ens.fr Tel: +351 210 480 100

Email: zmainen@neuro.fchampalimaud.org

# Methods

## **Viral transduction of dorsal raphe neurons and optical fiber implantation**

Adult (8–16 weeks) transgenic SERT-Cre [[1](#_ENREF_1)] or wild-type mice (C57BL/6 background, housed in a standard 12:12 hours light-dark cycle) were anaesthetized with isoflurane mixed with O_2_ (3% for induction and 0.5–1% for maintenance) or with ketamine xylazine (100 and 5 mg/kg) and placed in a stereotaxic apparatus (David Kopf Instruments). Lidocaine (2%) was injected subcutaneously before incising the scalp and exposing the skull. A craniotomy was drilled over lobule 4/5 of the cerebellum and a pipette filled with a viral solution (AAV2/1.EF1a.DIO.hChR2(H134R)-EYFP.WPRE.hGH, 10^13^ GC/mL, University of Pennsylvania) was lowered to the DRN (Bregma -4.4 to -4.7 mm AP, -2.8 to -2.9 mm DV) with a 34° angle toward the back of the animal (Fig. 1*A*). The viral solution (1–1.2 µL) was injected using a Picospritzer II (Parker) or a syringe pump (KDS 310 Plus, KD Scientific) at a rate of 0.05–0.06 µL/min. For optical fiber implantation, the skull was cleaned with H_2_O_2_ and covered with a layer of Super Bond C&B (Morita) before performing the craniotomy. An optical fiber (200 µm, 0.37 NA) housed inside a connectorized implant (M3, Doric Lenses) was inserted in the brain using the same 34° from the back, with the fiber tip positioned 200 µm above the core of the infection along the penetration axis. The craniotomy was covered with a drop of warm agarose gel (2%) and the implant was secured with dental acrylic (Pi-Ku-Plast HP 36, Bredent). The skin was stitched at the read and front of the implant and the animal was allowed to recover on a heating pad.

## ***In vitro* electrophysiological recordings and photostimulation of DRN neurons**

**Slice preparation, recordings and illumination.** DRN slices from male and female SERT-Cre mice aged 8-20 weeks (2–4 weeks post-infection) were prepared as follows. Mice were anesthetized with ketamine-xylazine (80 and 8 mg/kg) and perfused through the heart with 15 ml of cold (4°C) ACSF solution containing (in mM) 130 NaCl, 3.5 KCl, 1 NaH_2_PO_4_, 24 NaHCO_3_, 1 CaCl, 3 MgSO_4_, 10 glucose, saturated with 95% O_2_ and 5% CO_2_. The brain was then quickly removed and sliced (320 µm thickness) using a VT1200s vibratome (Leica). Slices were allowed to recover for 1 h at room temperature in an ACSF solution with a similar ionic composition as the slicing solution but containing (in mM) 3 CaCl_2_ and 1.5 MgSO_4_. This solution was used for recordings as well. Slices were next transferred to a recording chamber under an upright microscope (Olympus WI51) and cells were visualized by epifluorescence and IR-DIC videomicroscopy using a CCD camera (Hamamatsu Orca). For loose cell-attached recordings (n = 4 non-fluorescent and 28 fluorescent cells), pipettes (2–4 MΩ) were filled with ACSF, seal resistance was 30–90 MΩ, R_s_ was compensated to ~50% and cells were recorded in voltage clamp with 0 pA current injected. For whole cell recordings (n = 3 non-fluorescent and 6 fluorescent cells), pipettes (5–7 MΩ) were filled with (in mM) 126 K-gluconate, 4 KCl, 4 ATP-Mg, 0.3 GTP-Na_2_, 10 HEPES and 10 Creatine-phosphate (pH 7.25; 300 mOsm). Recordings were performed at 34°C using a Multiclamp 700B amplifier (Molecular Devices). Signals were filtered at 10 kHz, digitized at 20 kHz and analyzed with SpAcAn, a collection of IGOR Pro routines (www.spacan.net). Photostimulation was performed using a 465 nm LED coupled to a 200 µm, 0.37 NA optical fiber (Doric Lenses) and controlled by pCLAMP (Molecular Devices) via a universal LED driver (Mightex Systems). The optical fiber was held at 34° relative to the vertical axis and positioned above the slice using a PatchStar micromanipulator (Scientifica). The fiber tip was first placed against the slice within the field of view and then retracted axially by a known distance (0.5–2 mm).

**Calculation of incident irradiance.** For each cell, the incident irradiance was calculated by dividing the power measured at the fiber tip (measured with a PM100D powermeter, Thorlabs) by the area of the illuminated zone (Fig. S1 *A*). Given that the optical fiber was positioned above the slice at an angle, the illuminated area at the slice surface had an elliptic shape and could be calculated using the following equations:

The eccentricity $\varepsilon$ of the ellipse can be calculated from the half-angle of divergence of the cone of light ($\alpha$) and the angle of the fiber relative to the vertical axis ($\theta$):

$$\varepsilon=\frac{\cos\left( \alpha\right)}{\cos\left( \pi-\theta\right)} (1)$$

The half-angle of divergence $\alpha$ is defined by the fiber numerical aperture ($NA$) and the refractive index of the surrounding medium ($n$):

$$\theta= \sin^{-1} \left( \frac{NA}{n} \right) (2)$$

The semi-major ($a$) and semi-minor ($b$) axis of the ellipse are linked to the eccentricity by the following relation:

$$b=a\times\sqrt{1-\varepsilon^{2}} (3)$$

The semi-major axis can be calculated knowing the axial distance between the cone vertex and the slice ($z$):

$$a=z\times\frac{\cos\theta}{2}\times\left( \tan\left( \theta+\alpha\right)- \tan\left( \theta-\alpha\right) \right) (4)$$

Finally the area of the ellipse is given by the product$A= \pi\times a\times b$, which can be simplified as $A= \pi\times a^{2}\times\sqrt{1-\varepsilon^{2}}$ using$(3)$. By combining this expression with$(1)$, $(2)$ and $(4)$, we obtain:

$$A=\frac{\pi}{4}\times z^{2}\times\left( \cos\theta\right)^{2}\times\left( \tan\left( \theta+\alpha\right)- \tan\left( \theta-\alpha\right) \right)^{2}\times\sqrt{1-\frac{{(cos \alpha)}^{2}}{({\cos\left( \pi-\theta\right))}^{2}}}$$

**Photostimulation threshold.** The minimum amount of light required to activate ChR2-YFP cells was assessed by delivering brief pulses (6 ms) of increasing power at low frequency (1 Hz; Fig. 1*G*). Each cell responded in a nearly all-or-none fashion (Fig. 1*H*) and the photostimulation threshold was defined as the incident irradiance necessary to evoke a spike in half of the trials.

**Spike count versus pulse duration.** Firing profiles were characterized for different pulse durations. Light pulses of increasing duration were delivered in sweeps, each sweep consisting of a series of 0.1, 1, 2, 5, 10, 15, 30, 50, 100 and 1000 ms pulses separated by 1 s.

**Firing profiles for different photostimulation frequencies.** Cells were photostimulated with trains of 6 ms light pulses delivered at 1, 2, 5, 10, 20 and 50 Hz delivered in single sweeps, with an intersweep interval of 1 min.

## **Blue light propagation in the DRN**

The spread of blue light in the DRN was assessed *in vitro* using freshly dissected brains. Mice (n = 3) were anaesthetized using pentobarbital and decapitated. The posterior half of the brain was quickly dissected and embedded in 2% agarose gel. Blocks of tissue were then glued and immersed in the chamber of a vibratome (7000 smz 2, Campden Instruments), and trimmed in the parasagittal direction until the aquaduct was exposed. The hemi-cerebellum still attached to the brainstem, was carefully removed by sectioning the cerebellar pedunculi, and the block of tissue was immersed in a petri dish filled with PBS, placed under an upright microscope and imaged through a 5 x objective using a CCD camera (Retiga 4000R, QImaging) (Fig. S2 *A*). Light was delivered through a 200 µm 0.37 NA optical fiber positioned 110 ± 45 µm (n = 3) above the dorsal aspect of the DRN (Fig. S2 *B*) at the level of the plane of cut. Assuming that the relative pixel intensity scales proportionally with local irradiance, the intensity map provides an estimate of the spatial distribution of irradiance levels in the tissue (Fig. 2*B*). To assess how light spreads laterally in the DRN, the fiber was moved medio-laterally with 50 µm steps (Fig. S2 *B*–*F*). Because light is progressively more scattered as it penetrates into the tissue, the brightest pixel was not located at the DRN surface but 172 ± 23 µm below (n = 3). The relative pixel intensity was calculated along concentric circles centered on this point (circular profiles, Fig. 2*C*) and along the axis of the fiber (linear profiles, Fig. 2*D*).

## **Fluorescence mapping of ChR2-EYFP-expressing DRN neurons and electrophysiological recordings of local photoevoked activity *in vivo***

**Optical system.** Custom optrodes were assembled by gluing a cleaved 200 µm 0.37 NA multimode optical fiber (BFL37-200, Thorlabs) onto a platinum microelectrode (0.8–1.0 MΩ at 1 kHz, FHC). The optical fiber tip was positioned 300–500 µm above the microelectrode tip. Pulses of light were generated using a laser beam (473 nm, 100 mW DPSS laser, Laserglow) gated by a mechanical shutter (VS14S2ZM1, Uniblitz) and attenuated by a set of neutral density filters. The beam was passed through a 10:90 beamsplitter cube (BS025, Thorlabs) and the resulting 10% beam was collected by a photodiode (SM1PD1A, Thorlabs) for precise light pulse monitoring. The 90% beam was bounced on a dichroic mirror (T495LP, Chroma) and injected into the optical fiber using an aspheric lens (A240, Thorlabs). The fluorescence light travelling back in the fiber was passed through an emission filter (LP02-514-RU, Semrock) positioned after the dichroic mirror and focused onto the sensor of a custom cooled CCD camera using a N-BK7 plano-convex spherical lens (LA1255, Thorlabs). Fluorescence images were analyzed using MATLAB by measuring the average pixel intensity inside the fiber core. All fluorescence measurements were performed in the dark. The fiber intrinsic fluorescence (autofluorescence), assessed with the fiber in the air, accounted for 93 ± 11 % (n = 3) of the average fluorescence measured in the first millimeter of tissue.

The mechanical shutter yielded a minimal pulse duration of 6 ms. Power modulation was achieved using a set of 6 neutral density (ND) filters (transmissions of 0.1, 10, 26, 40, 68 and 81%, measured at 473 nm) positioned inside a filter wheel (FW102C, Thorlabs) and 3 additional filers added in the optical path (transmissions of 10, 32 and 50%). Laser power was set so that the power measured at the fiber tip was 5–6 mW using the 81% ND filter. The 0.1% ND filter was used for tissue fluorescence measurements (power at the fiber tip of around 10 µW). The 5 other filter wheel positions were used in combination with the 3 additional ND filters to produce 40 different power values for measuring the OLFP dependence on light intensity.

**Surgery and electrophysiological recordings.** Male and female SERT-Cre mice (2–4 weeks postinfection) or wild type littermates were anaesthetized with urethane (1.5 g/kg) and placed in a stereotaxic frame. Incision and craniotomy were performed as described for viral injection. The optrode was lowered in the brain using a 34° angled approach from the cerebellum, using an IVM micromanipulator (Scientifica). The electrophysiological signal was amplified (x1000) and filtered (0.1–10 kHz) by an 1800 AC amplifier (AM Systems), digitized (10 kHz) by a Micro1401-3 interface and acquired using Spike2 (Cambridge Electronic Design). Electrophysiological data were analyzed using IGOR Pro.

**Properties of the OLFP.** The positions of the optrode yielding a maximal fluorescence and OLFP (Fig. 2f) were assessed by fitting Gaussian functions to fluorescence and OLFP amplitude profiles. Fluorescence alone was mapped in 2 mice and combined fluorescence and OLFP profiles were obtained in 6 mice. The sensitivity of the OLFP to a 5-HT1A agonist was tested in 10 mice by sub-cutaneous injection of 375 µg/kg of 8-OH-DPAT (Tocris). The dependence of the OLFP on the irradiance at the fiber tip was tested by delivering 6 ms light pulses of various intensities at 1 Hz (40 different irradiance values ranging in average from 0.64 ± 0.95 to 191.94 ± 5.73 mW.mm^-2^, 10 repetitions per intensity, n = 9). The effect of repeated photostimulation was assessed using 12 s trains of light pulses (6 ms) delivered at 0.5, 1, 2, 5, 10, 20 and 50 Hz in single sweeps, with an intersweep interval of 1 min. The average OLFP amplitude and peak latency were calculated over the last 4 s of each train. The OLFP recovery rate was assessed using sweeps consisting of a 10 Hz, 5 s trains of light pulses (6 ms) followed by a single test pulse at a variable interval after the train (δt), with an intersweep interval of 30 seconds (Fig. 2*K*). The recovery rate was fitted using an exponential function. Full recovery was observed after 7.6 s.

## **Von Frey test**

In the von Frey test, a series of calibrated Nylon filaments (von Frey filaments, Bioseb) of ascending stiffness are applied to the plantar surface of each hind paw (avoiding the toes, heel and pads) while monitoring the animal’s response. The main response is paw withdrawal but other responses can be observed such as fingers extension. The test allows repeated measures over time with minimal habituation and therefore is well suited for within-animal comparisons and response averaging. Once bent, each filament exerts a constant pressure on the skin (see Table 1 for a chart of theoretical pressures), and repeated measures with the same filament allow calculating the response probability for this filament.

A total of 34 mice were tested: 17 SERT-Cre mice infected with the Cre-dependent viral vector and 17 wild type littermates (control group) infected with the same virus. Prior to testing, animals were allowed to habituate to the testing box (a 9 x 7 x 14 cm Plexiglas box placed on an elevated metal mesh platform) 5 minutes per day for 5 days. During these habituation sessions, the animals were plugged to a fiberoptic patchcord (Doric Lenses), and the 4.0 g filament was applied 5 times per hind paw. In the testing phase, each animal was subjected to 3 to 4 testing sessions (with a maximum of 1 session per day). All sessions took place during the light period of the light-dark cycle, between 10:00 a.m. and 5:00 p.m. Each session was divided in 3 blocks, designed to test the animal’s sensitivity prior to (“baseline”), in conjunction with (“stim”) and after (“recovery”) photostimulation (Fig. 3*B*). In each block, filaments ranging 0.4–8 g were applied consecutively in an ascending fashion (each filament was applied 5 times successively to the right and left hind paws), yielding one psychometric curve (response probability versus filament) per block (for each curve the left and right response probabilities were averaged). The threshold was taken as the interpolated filament value corresponding to a response probability of 0.5. The “stim” and “recovery” blocks were separated by a 5 minutes delay. In the “stim” condition, filament application was restricted to the last three quarters of a 12 s photostimulation train (10 ms, 20 Hz, 318 mW.mm^-2^ at the fiber tip; Fig. 3*C*). The 3 s delay between photostimulation onset and first filament application are meant to allow the establishment of a potentially slow serotonergic neuromodulatory mechanism. The stimulation parameters were chosen to produce a saturating effect based on our *in vitro* and *in vivo* characterization. Light pulses were generated using a custom system based on an acousto-optic modulator (AOM). AOMs have combined high-precision power modulation and shuttering capabilities. Unlike mechanical shutters, AOMs operate in a totally silent way (AOMs use inaudible acoustic frequencies, typically around 100 MHz) and are therefore well suited for experiments with behaving animals. All sessions were videotaped and analyzed offline. The experimenter was blind to the genotype of the mice throughout both the testing and offline scoring phases.

# Supplementary table

**Table S1.** Von Frey filaments force scale.

| Filament target force (g) | Target force (mN) | Theoretical pressure  (g.mm^-2^) |
| --- | --- | --- |
| 0.16 | 1.6 | 8.77 |
| 0.4 | 3.9 | 16.1 |
| 0.6 | 5.9 | 18.4 |
| 1.0 | 9.8 | 24.4 |
| 1.4 | 13.7 | 27.9 |
| 2.0 | 19.6 | 27.4 |
| 4.0 | 39.2 | 40.3 |
| 6.0 | 58.8 | 52.6 |
| 8.0 | 78.4 | 61.7 |
| 10.0 | 98.0 | 68.3 |

# Supplementary figure legends

**Figure S1**. Photostimulation of DRN 5-HT neurons *in vitro*. (*A*) Calculation of the mean incident irradiance. The optical fiber was positioned at an angle (θ) above the slice, with a known axial distance from the slice surface. The cone of light exiting the fiber formed an ellipse onto the slice, whose area can be calculated based on the axial distance between the cone vertex and the slice (z), the fiber angle θ and the divergence half-angle (α) of the fiber (see Methods). (*B*) Average spike probability per pulse as a function of pulse duration for the two irradiance tested (twice the PT, dotted line, n = 17, and maximal irradiance delivered by the LED, dashed line, n = 11). Most cells fired reliably (spike probability > 0.80) for pulses longer than 5 ms at twice their PT (n = 16/17), and for pulses longer than 1 ms at higher irradiance (n = 10/11, 5.1 ± 3.7 mW.mm^-2^). (*C*) Example traces from two cells recorded in the whole cell configuration and illuminated with a 1 s pulse. Although the two cells had similar input resistances (629 and 565 MΩ) and photostimulation thresholds (0.094 and 0.128 mW.mm^-2^), one displayed rapid inactivation (bottom) while the other one did not (top). (*D***–***E*) Firing adaptation during 5 s trains of repeated photostimulation at fixed frequencies, at two irradiance levels: twice the PT (*D****_1_*–***D****_3_***) and maximal irradiance produced by the LED (~ 5 mW.mm^-2^, *E_1_*–*E_3_*). The graphs show the average spike probability per pulse as a function of time (*D_1_* and *E_1_*, bin = 1 s) and as a function of the stimulation frequency (*D****_2_*** and *E****_2_***) for the first and last time bins (black and white arrowheads), as well as the time course of the average firing rate for various stimulation frequencies (*D****_3_*** and *E****_3_***). The color code in *D****_1_*** and *E****_1_*** is the same as *D****_3_*** and *E****_3_***. The 0.85 spike probability is marked in *D****_2_*** and *E****_2_*** by a horizontal dotted line. The numbers of cells averaged for panels *D****_1_****–D****_3_*** are n = 29, 28, 25 and 18 cells for 1–2, 5–10, 20 and 50 Hz respectively. The numbers of cells averaged for panels *E****_1_****–E****_3_*** are n = 14 and 13 cells for 1–20 and 50 Hz respectively.

**Figure S2**. Light propagation in the DRN. (*A*) Picture of a fresh brain hemisected in the sagittal plane (see Methods). The DRN corresponds to the grey matter localized inside the dotted white square. (*B*) Schematics representing the portion of tissue delimited by the white square in A and the optical fiber positioned above it. The spread of light in the tissue was estimated along the axis of the fiber (Z) and along the antero-posterior axis (AP). The fiber was moved medio-laterally (ML) in order to assess how light diffused laterally in the tissue. (*C*) Average dimensions of the volume of tissue receiving more than 10% (light grey) and 2% (dark grey) of the maximal intensity along Z and the AP axis. (*D*–*F*) Color coded pictures representing the relative pixel intensities for various ML positions of the fiber for the 3 brains examined. The fiber was moved every 50 µm in the ML axis over 500 µm.

# References

1. Gong, S., et al., *Targeting Cre recombinase to specific neuron populations with bacterial artificial chromosome constructs.* J Neurosci, 2007. **27**(37): p. 9817-23.
